# Parasitic fauna of domestic cavies in the western highlands of Cameroon (Central Africa)

**DOI:** 10.1186/s12917-015-0605-4

**Published:** 2015-11-26

**Authors:** Marc K. Kouam, Felix Meutchieye, Terence T. Nguafack, Emile Miegoué, Joseph Tchoumboué, Georgios Theodoropoulos

**Affiliations:** Department of Animal Production, Faculty of Agronomy and Agricultural Sciences, PO BOX 122, Dschang, Cameroon; Department of Anatomy and Physiology of Farm Animals, Faculty of Animal Science and Aquaculture, Agricultural University of Athens, 75 Iera Odos St, Votanikos, Athens, 11855 Greece

**Keywords:** Guinea pigs, Parasites, Ectoparasites, Cameroon

## Abstract

**Background:**

Domestic cavies (*Cavia porcellus*) are increasingly reared in rural areas of Cameroon for meat and income generation. Unfortunately, health constraints due to various pathogens including parasites stand as one of the major obstacles to the development of cavy industry in the country. The main objective of this study was to investigate the species of gastrointestinal parasites in cavy husbandry in the western highlands of Cameroon and to detect external parasites in those animals affected with dermatological disorders.

**Methods:**

Pooled fecal samples were collected from 62 privately-own farms, as well as individual fecal samples from 21 animals at the Teaching and Research Farm of the University of Dschang, and examined for parasite eggs and oocysts/cysts. Ectoparasites were also collected from cavies and identified.

**Results:**

The overall infection rate with both helminthes and arthropods was 40.3 %. Ectoparasites were found in 19 out of 62 farms (30.6 %) while 12.9 % of farms were infected with helminthes. Eggs of *Graphidium strigosum* (8.1 %), *Trichostrongylus* sp. (3.2 %) *and Paraspidodera uncinata* (3.2 %) were found at farm level. Oocysts of *Eimeria caviae* and eggs of *Paraspidodera uncinata* were found in 14.3 and 9.5 % of examined animals respectively. Concerning ectoparasites, *Cordylobia anthropophaga* and *Pulex* sp. were observed in 25.8 % and 6.6 % of farms respectively.

**Conclusion:**

The parasites are apparently composed of host-specific species in the original habitat (South America) and species acquired later from other mammals. These parasites are either deleterious to cavy health or zoonotic. Preventive measures should be put in practice to avoid their presence on farms due to their harmful effect on cavy rearing.

## Background

Domestic cavies commonly known as guinea pigs (*Cavia porcellus*) are increasingly becoming popular among farmers in the western highland of Cameroon, as an additional source of income and meat [1]. Mostly used as pet or laboratory animal all over the world [2], cavies are being reared essentially as source of protein in Cameroon following the incitation to diversify the source of meat by FAO and the Cameroon Ministry of livestock [3, 4]. However, the interest in this livestock species is hampered by many challenges including diseases, inadequate nutrition, poor management practices, mice cannibalism on the pups, and predation by dogs and cats [5]. Some works were done to address inadequate nutrition issues [6, 7], poor management practices [3] but in regards to health problems, much effort is needed to be undertaken. Parasitic infections have been reported as a common health problem in cavies in various areas of the world such as Europe [2, 8, 9], South America [10, 11], and Africa [12].

Regardless of the breeding system, cavies naturally harbor a wide range of parasites including arthropods, helminthes, and protozoa [11, 13]. In western Cameroon highlands region where cavies are mostly kept in a semi extensive system, the basic information on their parasitic fauna is lacking. The guinea pig originated in the Andes of South America [14] and has become a world-wide distribution as pet and laboratory animal. In South America and some other regions in the world guinea pigs are kept as food source [14]. Parasites of guinea pigs in their original homelands and those kept as laboratory animals are well known but little is known about the parasite fauna of the host species in semi-extensive or intensive conditions in other regions. Therefore, the main objective of the present study was twofold:

1. to investigate the species of gastrointestinal parasites in cavy husbandry in the western highlands of Cameroon and
2. to detect external parasites in those animals affected with dermatological disorders.

## Methods

### Study area and farms

The study was carried out between March 2013 and February 2014 in the western highlands of Cameroon, an agro-ecological zone covering the North West and west regions of the country, located between latitude 5°20’-7° North and Longitudes 9°40’-11°10’ East. The region is characterized by high relief and the climate is of Sudano-Guinean type. The region has one rainy season, which lasts from mid March to Mid November and one dry season from mid November to mid March. Humidity varies from 80 to 98 %. Annual precipitation ranges from 1500 to 2500 mm while minimum and maximum temperature are 10 °C and 34 °C respectively [15]. Originally, the vegetation of this region was of the savannah type but over the years due to intense crops production and animal rearing it has been transformed to semi-degraded or degraded forest type. Nevertheless, the original vegetation can be observed in certain parts of the region which is characterized by an increasing population growth, one of the highest in the country.

The study was conducted both in privately-owned farms and the Teaching and Research Farm of the University of Dschang (TRF), all located in the Menoua Division. Private farms are owned by small scale farmers also having rabbits, sheep, goats, or local fowl breeds. The housing system in the private farms is either the raised floor system or in most cases, the traditional free kitchen roaming system; in this latter system, cavies share the kitchen floor with the local fowl and/or small ruminants, and feed on kitchen waste and forages. Forages are harvested for free as part of the natural vegetation in the compound. Regarding the TRF the housing system is the deep litter system in lodges and animals are fed concentrates and forages. The breeding system is semi-extensive in privately –owned farms, and intensive in the TRF. In the area, 45 % of farmers keep cavies as additional source of income, 30 % for manure production for backyard crop production, 20 % for meat, and 5 % as pet [1].

### Ethical approval

This research did not involve experiments on animals. Feces and external parasites collected on animals were performed in accordance with all applicable international guidelines for the care of animals. Farmers accepted to participate in the study by granting oral informed consent, and allowed the collection of data from their cavies.

### Study design and sample collection

There is no central registry of farms in Cameroon so private farms were located and visited using a snowball sampling technique whereby a farmer, when located helped to locate the next farm and so on. For private farms where all animals are gathered in the same space, a pooled fecal sample per farm was collected into a suitable container containing 10 % formalin, and stored at room temperature until analysis. When the total number of animal per farm was greater than five, at least five animals were allowed to feed into a large, clean bucket and the feces were collected directly from the bucket soon after defecation. When the number was less than five, all the animals were sampled. For TRF, all the lodges within the building were visited, and two cavies per lodge (one male and one female) were randomly sampled. Each cavy was allowed to feed into a box, and the fecal sample was collected soon after defecation. Fecal samples were taken the same day to the Laboratry of Animal Health, Department of Animal Productions (University of Dschang) and analyzed immediately.

Whether at the privately-owned farm or at the TRF, cavies were carefully checked for any sign of ectoparastism. The ectoparasites from suspicious animals (with hair loss or limping) were brushed off or pulled out manually onto a white cloth, then preserved in 80 % ethanol and stored at room temperature until identification.

### Processing of fecal samples

Faecal samples were analyzed qualitatively and quantitatively using the saturated salt solution (NaCl) as flotation fluid. The simple flotation method was used to detect the parasite eggs and oocysts which were identified microscopically based on morphology and size [16]. The Modified Mc Master [17] test, with a sensitivity of 50 eggs per gram of feces (epg) was used to estimate the parasitic burden in the individual cavy fecal samples. This burden was used to determine the degree of parasite aggregation. Heavy eggs were screened using the simple sedimentation test, as described by Zajac and Conroy [17]. Slides were mounted and examined at 100 and 400 magnification.

### Identification of ectoparasites

Ectoparasites collected were identified to the genus and species level based on their morphology, using a stereomicroscope (up to 100 × magnifications) and following the identification key provided by Erzinclioglu [18] for skin larva, and by Zajac and Conroy [17] for fleas. The larvae were distinguished and identified from other species of the genus *Cordylobia* Grunberg, by focusing on the posterior spiracular apertures, the spines and the mouth hook. Fleas were identified by focusing on the genal and pronatal comb, the front marging of head, the mesopleuron, and the ocular bristle.

### Statistical analysis

Data were analysed using desciptive statistics. The prevalence and the degree of parasite aggregation (k) were estimated as described by Permin *et al*. [19].

## Results

### Infection rate at farm level

A total of 62 farms were investigated. The number of animals per farm varied from 2 to 18.

Animals were found to be infected with at least 3 helminth species and 2 ectoparasitic species. The overall infection rate of helminth infection was 12.9 % (8 out of 62) (95 % confidence interval, CI: 5.7–23.9). The specific infection rates were 8.1 % (5 out of 62), 3.2 % (2 out of 62) for *Graphydium strigosum*, *Trichostrongylus sp.* and *Paraspidodera uncinata* each respectively (Table 1). One farm was co-infected with *G strigosum* and *Trichostrongylus* sp.

**Table 1:** Gastrointestinal parasites and ectoparasites in domestic cavy farms in the Menoua division, Cameroon

Species	Number of farms examined	Number of farms positive	Percentage (%)	95%CI^a^
*Graphidium strigosum*	62	5	8.1	2.7–17.8
*Trichostrongylus* sp.	62	2	3.2	0.4–11.2
*Paraspidodera uncinata*	62	2	3.2	0.4–11.2
Overall helminthes	62	8^c^	12.9	5.7–23.9
* Cordylobia anthropophaga*	62	16	25.8	15.5–38.5
* Pulex* sp.	61^b^	4	6.6	1.8–15.9
Overall arthropods	62	19^d^	30.6	19.6–43.7
Overall infection	62	25	40.3	28.1–53.6

a: Confidence interval; b: Data on *Pulex* sp. was missing on one farm; c: One farm was co-infected with *G strigosum* and *Trichostrongylus* sp.; d: One farm was co-infected with *C. anthropophaga* and *Pulex* sp

**Fig. 1:**
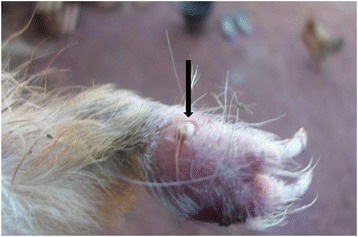
Lesion of *Cordlylobia anthropophaga* on a domestic cavy foot. Arrow shows a part of the white body of the larva, the spiracle, in the center of the lesion

**Fig. 2:**
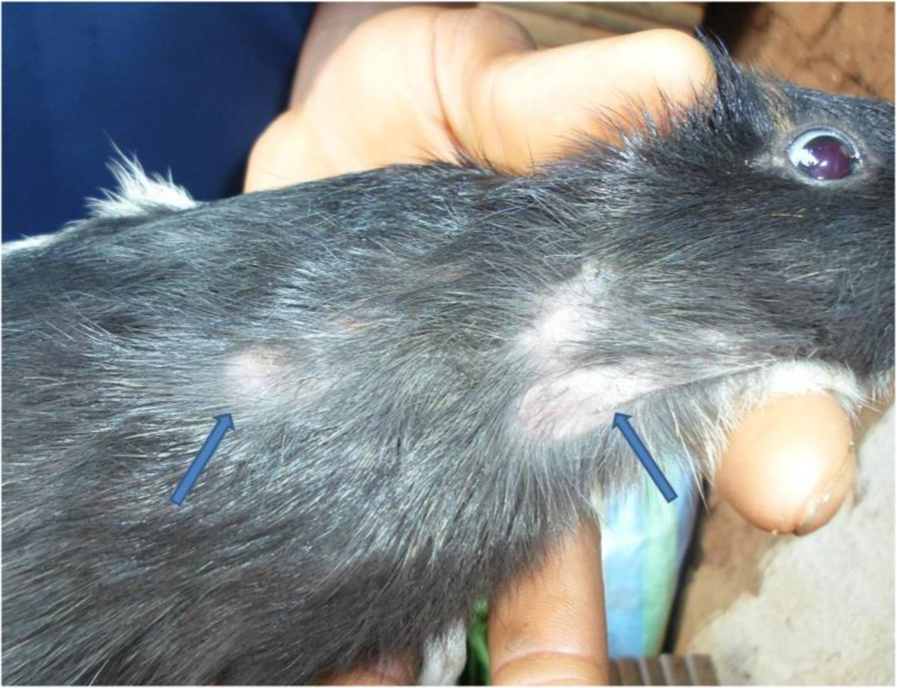
Lesions of flea bite in a domestic cavy (see arrows)

Ectoparasites were found in 19 out of 62 farms (30.6 %). Larvae were identified as *Cordylobia anthropophaga* (Blanchard), the tumbu fly larvae. Animals infested with this parasite had difficulties to walk and run properly. Any area at the ventral part of the animal could be infested but lesions were predominant at the legs (Fig. 1) and secondly around the stem of the tail. This parasite was present in 25.8 % (16 out of 62) of farms visited. Fleas were all identified to genus level as *Pulex* sp. The mesopleuron of the specimen examined was not divided by a vertical thickening, indicating the occurrence of the genus *Pulex* rather than the closely related genus *Xenopsylla*. Difficulties to locate the ocular britle made the identification to species level quite impossible. The farm infection rate was 6.6 % (4out of 62) (Table 1). Heavy infestation with this flea was featured by a serious hair loss all over the body (Fig. 2). One farm was co-infected with *C. anthropophaga* and *Pulex* sp. The overall infection rate with both helminthes and arthropods was 40.3 %.

### Infection rate at the teaching and research farm

Gastrointernal parasites were detected in 4 out of 21cavies examined (19 %) (95 % CI: 5.4–41.9). *Eimeria caviae* was detected in 3 out of 21 animals (14.3 %) while *Paraspidodera uncinata* was found in 2 out of 21 animals (9.5 %) (Table 2). There was a co-infection with both parasites in a single animal. The degree of parasite aggregation (k) for *E. caviae* was 147.23 and for *P. uncinata* was 0.66.

**Table 2:** Gastrointestinal parasites of domestic cavies at the Teaching and Research Farm

Species	Number of animals examined	Number of animals positive	Percentage (%)	95 % CI^a^
*Eimeria caviae*	21	3	14.3	3–36.3
*Paraspidodera uncinata*	21	2	9.5	1.2–30.4
Overall infection	21	4^b^	19.6	5.4–41.9

a: Confidence interval; b: There was a co-infection with *E. caviae* and *P. uncinata* in a single animal

No heavy eggs were found using the sedimentation method.

## Discussion

The number of animals per farm was low for the owners to be qualified as “farmers”. The fact is that people are being encouraged by the government to rear domestic cavies, so that even a beginner with a small number of animals is regarded as a farmer. This is the first report on parasitism in mini-livestock cavies in the werstern highlands of Cameroon, and also in the entire country. It is also the first report of *Cordylobia anthropophaga* infection in cavies in the country. In total, six parasitic species were observed in this study. These parasites are either deleterious to cavy health and/or zoonotic.

Three gastrointestinal parasite species from cavies were found on farms. Cavies can be host to many parasites species occurring both naturally and experimentally. As pet, meat or laboratory animal, cavies have been found infected with helminthes such as the nematode *Paraspidodera uncinata* and *Trichostrongylus colubriformis* [11]*,* the cestode *Monoecocestus* parcitesticulatus and *Hymenolepis* (or *Rodentolepis*) *nana* [20, 21], and the Trematode *Fasciola* [22]. Experimentally, cavies were shown to be susceptible to a huge range of helminthes and protozoa including *Trichinella* [23], *Leishmania* [24], *Trypanosoma* [25], *Cryptosporidium* [26] and others. Therefore, much more parasitic infections were expected in the farms, especially when livestock husbandry in rural areas is carried out in mixed species systems, and forages fed to animals are wet in most cases. Use of most sensitive detection techniques will probably reveal the presence of other cavy gastrointestinal parasites not found in the present work.

The three helminthes found in this study were *Graphydium strigosum, Trichostrongylus* sp., and *Paraspidodera uncinata*.

The genus *Graphidium* has not been described in cavies up to now. In this study, *G. strigosum* was found in up to 5 farms investigated. It is known that domestic cavies are coprophageous [27, 28] and there is a possibility that this parasite may be spurious since adult parasites were not demonstrated. *G. strigosum* is known to occur in rabbit serving as natural host [29]. It could be hypothesized that *G strigosum* was acquired as a result of the breeding conditions of both rabbits and cavies. In the study area, rabbits and cavies are most often kept together indoor, close to man in the kitchen, with rabbits housed in cages and cavies moving free on the floor. The genus *Trichostrongylus*, notably *T. colubriformis* has been previously described in domestic cavies [11, 30] showing a prevalence of 2 % [11]. In this study, *Trichostrongylus.* sp. was found in only two farms (3.2 %). Though the sampling unit (cavy in the previous study and farm in the present study) in both studies is different, the level of infection with this genus in both cases is low. Despite the low level of infection, care (regular deworming, full implementation of hygiene on the farm, supply of dried rather than wet forages to animals, and others) should be taken to expel these parasites from farms because some species such as *T. retortaeformis* are known to cause serious damages in the intestinal mucosa of the host [31]. It is necessary to describe this parasite to species level for two reasons: 1) some species of the genus *Trichostrongylus* are very pathogenic and therefore may cause serious problems in cavy farming; 2) since farmers in rural areas also keep ruminants usually harboring a wide range of *Trichostrongylus* species, there is a possibility of cross infection between cavies and ruminants that may contribute to parasite survival. *P. uncinata is a* specific parasite that has been found in wild cavies (*Cavia aperea aperea*) [32] and extensively described in domestic cavies [8, 11, 13, 20] in different breeding systems and conditions. In this study, *P. uncinata* was found both in conventional rural farms and the teaching and research farm confirming that this parasite is a ubiquitous species, with large geographic distribution, that can adapt to various breeding conditions. An important observation in the present study was that *P. uncinata* eggs in the examined cavies were aggregated in a few individuals, which is indicative of overdispersion (k < 1). Overdispersion is the most common form of frequency distribution of parasitic communities in nature [33] and is generated by variation between individual hosts in their exposure to parasite infective stages and by differences in their susceptibility after an infectious parasite has been encountered [34].

Coccidiosis due to *E. caviae* is another problem in domestic cavy worldwide [35, 36]. A recent study in Italy [8] showed an infection rate of 10 %, which is comparable with the rate obtained in this study (14.3 %). Though the infection rate was low, the infection was severe with greater than 10000 oocysts per gram of feces in a single animal. This might be related to the poor hygienic conditions associated with the husbandry practices of the geographic origin of the animal purchased. Indeed cavies purchased from different sources for use at the TRF are often introduced in the flock without any quarantine period. No overdispersion (k > 1) was observed in the distribution of *E. caviae* indicating a uniform exposure and/or susceptibility of the animals to the parasite.

The ectoparasites found were *C. anthropophaga* and *Pulex* sp. *C. anthropophaga* has been described in a wide range of mammals including man and domestic animals (Fujisaki *et al*., 2008; Ogo *et al*., 2009) [37, 38]. Since the adult fly is attracted by urine smell, the high infection rate of farms may be attributed to the poor hygienic conditions of the living space characterized by accumulation for days of kitchen waste and forage leftovers. Presence of this parasite in domestic cavies is a real obstacle in cavy keeping because an animal with infestation at the leg is disabled and cannot properly compete for food. Another consequence of the presence of this parasite is the risk of zoonosis because the infested cavies stand as perfect reservoirs towards this parasite which also occurs in humans.

Various flea species from different genera occur in cavies [10, 39]. *Pulex irritans*, also known as human flea has been reported in domestic animals such as cats and dogs but can also be found in cavies [10, 39, 40]. Specimen found in this study were described as *Pulex* sp., but urgently need to be identified to species level with improved tools and techniques (including molecular techniques) due to the close proximity of cavies with other domestic animals in addition to humans. Measures (good hygienic practices, rapid treatment of infested animals, practice of quarantine) should be taken to prevent infestation with this insect on the farm, since infested animals showed hair loss probably as a consequence of intense irritation and pruritus.

Apart from *P. uncinata* found in rural farms and the TRF, other organisms were detected for only one farm type, with helminthes and ectoparasites in rural farms and coccidian in the TRF. This could be the result of poor breeding conditions in rural areas more conducive to the development and dissemination of parasites than in the TRF.

Globally, the infection rate was low but since the government is encouraging cavies farming, this rate will certainly increase as the result of the involvement of many rural populations in cavies farming. One of the consequences of this massive farming will probably be the spreading of zoonotic parasitic diseases and other parasitic infections. For a successful future in cavies farming, it is the government’s responsibility to train dedicated veterinarians and educate cavies’ keepers on their duties.

## Conclusion

Three groups of parasites including helminthes, protozoa and insects occur in domestic cavies reared in the western highlands of Cameroon. The parasites are apparently composed of host-specific species in the original habitat (South America) and species acquired later from other mammals. Identification of *Pulex* sp. and *Trichostrongylus* sp. to species level is necessary for discussion of their pathogenicity, their zoonotic potential and the *Pulex* transmission ability of bacterial diseases to humans. Preventive measures should be implemented to keep these parasites away from the farm in order to avoid their harmful effect on cavy keeping. Further studies using improved diagnostic tools and techniques, with increased sample size still need to be undertaken in order to provide a full parasite fauna occurring in cavies reared in the two breeding systems currently in place in Cameroon.

## Abbreviations

BecA: Biosciences Eastern and Central Africa
CSIRO: Commonwealth Scientific and Industrial Research Organization
FAO: Food and Agriculture Organization of the United Nation
ILRI: International Livestock Research Institute
NaCl: Sodium chloride
TRF: Teaching and Research Farm of the University of Dschang
